# Transformations and Summations for Bilateral Basic Hypergeometric Series

**DOI:** 10.1007/s40065-026-00622-7

**Published:** 2026-06-20

**Authors:** Howard S. Cohl, Michael J. Schlosser

**Affiliations:** 1 Applied and Computational Mathematics Division, National Institute of Standards and Technology, Mission Viejo, 92694 CA, USA; 2 Fakultät für Mathematik, Universität Wien, Oskar-Morgenstern-Platz 1,1090, Vienna, Austria

**Keywords:** 33D15, 33D45

## Abstract

We review and derive transformation and summation formulas for bilateral basic hypergeometric series. Our study focuses on consequences of certain bilateral extensions of two important results by Bailey, namely a transformation for very-well-poised $_8\!W_7$ series in terms of two balanced $_4\phi _3$ series, and a transformation connecting three $_8\!W_7$ series. Two rather recently discovered transformations of bilateral basic very-well-poised ${}_8\!\Psi _8$, one by Zhang and Zhang, the other by Wei and Yu, serve as the starting point of our investigations. From these transformations we work out interesting special cases that were not considered before, including explicit bilateral quadratic and cubic summations. We further explicitly record noteworthy lower-level transformations derived by taking suitable limits and deduce more transformations by exploiting the symmetry of the parameters in the series.

## Introduction

The theory of basic hypergeometric series [5] is rich in identities. This theory arose out of an interest to extend classical results for hypergeometric series to that for the *basic* (or *q*-)case. The first systematic development of the theory was undertaken by Heine in the 1840 s, but only some decades later, in the first half of the 20th century, its development attracted broad interest, with researchers such as Jackson, Bailey, Watson, Slater, and others, being leading pioneers.

While numerous identities (summation and transformation formulas) for unilateral basic hypergeometric series exist, not so many identities are known for *bilateral* basic hypergeometric series, the list of closed form summations essentially being limited (in one variable, i.e., for single series) to Ramanujan’s $_1\!\psi _1$ summation and Bailey’s very-well-poised $_6\psi _6$ summation and their special cases (such as Jacobi’s triple product identity, and the quintuple product identity), apart from some other summations (such as those of *q*-Karlsson–Minton type which even hold for a general number of parameters, or, on the contrary, summations for some highly specialized bilateral basic hypergeometric series, such as those of Rogers–Ramanujan type [13]).

This paper is devoted to the study of identities for bilateral basic hypergeometric series (in one variable), focusing on identities containing series up to the level of $_8\!\psi _8$ basic hypergeometric series. (For identities on the level of $_{10}\psi _{10}$ series we merely review the general ones considered by Slater. However, they do not appear to be bilateral extensions of Bailey’s 4-term very-well-poised ${}_{10}\!W_9$ transformations or of relevant special cases of them, such as Bailey’s 3-term very-well-poised $_8\phi _7$ transformations.) The identities we feature are all deduced from transformation formulas for $_8\!\psi _8$ series obtained by Zhang and Zhang [19] (we correct their main result) and by Wei and Yu [18]. On one hand, we hope that the compilation of identities we give, having everything at one place, is useful. On the other hand, we list some particularly nice consequences that were not considered explicitly before, such as an interesting cubic summation, or some quadratic summations; these are derived by specializing the $_8\!\psi _8$ transformations and combining them with other known results.

The remainder of this introductory section is devoted to some preliminaries regarding notation. In Sect. 2 we recall some important identities involving unilateral basic hypergeometric series that we make use of. In Sect. 3 we turn to bilateral basic hypergeometric series with a focus on various transformations satisfied by them. In Theorem 3.3, we have presented a corrected version of the Zhang–Zhang transformation of a very-well-poised ${}_8\!\psi _8$ [19, Theorem 6] which is a bilateral extension of Bailey’s transformation [5, (17.9.16)] of a nonterminating very-well-poised ${}_8\!W_7$ in terms of a sum of two balanced $_4\phi _3$ series. In the new Theorem 3.5, we give a $q\rightarrow 1^{-}$ limit of the Zhang–Zhang transformation which provides a transformation of a very-well-poised ${}_7\!H_7$ in terms of a ${}_4\!H_4$ and two balanced ${}_4F_3$’s. By starting with Theorem 3.3, we obtain in Corollary 3.8 a new cubic summation formula involving a very-well-poised ${}_8\!\psi _8$ series and a ${}_2\phi _1$ series with base $q^3$. The specialization of Corollary 3.8 to the unilateral case (a cubic summation involving a very-well-poised ${}_8\!\phi _7$ series and a ${}_2\phi _1$ series with base $q^3$) already appears to be new and is given as Corollary 3.9. In Corollary 3.12 we present a new transformation of a very-well-poised ${}_8\!W_7$ in terms a ${}_4\psi _4$ and two balanced $_4\phi _3$’s. In Theorem 3.13 we revisit the Wei–Yu transformation of a very-well-poised ${}_8\!\psi _8$ in terms another very-well-poised ${}_8\psi _8$ and a very-well-poised ${}_8\!W_7$ [18, Theorem 3]. In Corollaries 3.16, 3.18, 3.19 we present new nonterminating summation formulas for very-well-poised ${}_8\!\psi _8$’s. Corollaries 3.18, 3.19 are bilateral extensions of the nonterminating very-well-poised ${}_8\!W_7$ summations [5, (II.16), (II.18)]. By equating the Zhang–Zhang and Wei–Yu transformations, we give a new transformation of a very-well-poised ${}_8\!\psi _8$, a ${}_4\!\psi _4$, an ${}_8\!W_7$, and two balanced $_4\phi _3$’s. Note that in [18] the authors only realized that their Theorem 3.13 extended Bailey’s ${}_6\!\Psi _6$ summation (see Remark 3.14). Other than this, all consequences of the Wei–Yu transformation which appear in this manuscript appear to be new.

In Theorem 2.1, we derive a sequence of transformation formulas by starting with Bailey’s transformation of a very-well-poised ${}_8\!W_7$ in terms of a sum of two balanced $_4\phi _3$’s. This interesting sequence of transformations was studied in [17] (see in particular [17, Figure 2]). van de Bult and Rains (2009) start from Bailey’s four-term transformation formula for nonterminating ${}_{10}\!W_9$. Starting with Bailey’s transformation of a very-well-poised ${}_8W_7$ in terms of a sum of two balanced $_4\phi _3$’s, the first limit gives a transformation of a ${}_7\!W_6^1$ series to a special ${}_3\phi _2$ series (24). The second limit gives the transformation of a ${}_6\!W_5^2$ to a ${}_2\phi _1$ with an arbitrary argument and general parameters. The extension of this transformation to bilateral basic hypergeometric series was discovered by Bailey (37); it relates a ${}_6\!\Psi _6^2$ to a ${}_2\!\psi _2$. One of the goals of this paper is to study a scheme of transformations for bilateral basic hypergeometric series that is analogous to that for the unilateral transformations of Bailey-type, where the elements in the scheme are connected by taking limits.

Consequences of the featured transformation formulas are given in Sects. 4 and 5. Since the corrected Zhang–Zhang result provides a bilateral extension of Bailey’s transformation (23), we utilize that result in Sect. 4 to deduce bilateral lower-level transformations by suitably taking limits. In Sect. 5, we derive further transformation formulas, not by taking limits but by starting with the transformation formulas derived in Sect. 4 and exploiting the symmetry in the parameters. While the transformation formulas in Sects. 4 and 5 are all routine consequences of more general results, we record them explicitly as we believe that it is useful to have them available in one place. As far as we are aware, they have not been stated explicitly before (with exceptions, such as the very useful Corollary 5.14), and appear for the first time in this paper.

### Preliminaries

#### Definition 1.1

We adopt the following conventions for succinctly writing elements of multisets. To indicate sequential positive and negative elements, we write

$$ \pm a:=\{a,-a\}. $$

We also adopt an analogous notation

$$ z^{\pm }:=\{z,z^{-1}\}. $$

We adopt the following set notations: $\mathbb {N}_0:=\{0\}\cup \mathbb {N}=\{0, 1, 2,\ldots \}$, and we use the sets $\mathbb {Z}$, $\mathbb {R}$, $\mathbb {C}$ which represent the integers, real numbers, and complex numbers respectively, ${{\mathbb {C}}^*}:={{\mathbb {C}}}\setminus \{0\}$. Define the set $q^{\mathbb {Z}}:=\{q^k:k\in \mathbb {Z}\}.$ The *q*-shifted factorial is given by

1 $$\begin{aligned} (a;q)_n:=(1-a)(1-qa)\cdots (1-q^{n-1}a),\qquad n\in \mathbb {N}_0, \end{aligned}$$

and one further defines

2 $$\begin{aligned} (a;q)_\infty :=\prod _{n=0}^\infty (1-aq^{n}), \end{aligned}$$

where $|q|<1$. Using this infinite product, one can extend the *q*-shifted factorial to arbitrary complex index *b*, by defining

3 $$\begin{aligned} (a;q)_b:=\frac{(a;q)_\infty }{(q^ba;q)_\infty }. \end{aligned}$$

In particular, when dealing with bilateral series we will frequently use the *q*-shifted factorial with the index *b* being a negative integer, say, $b=-n$ where $n\in \mathbb {N}$, in which case we see that

4 $$\begin{aligned} (a;q)_{-n}=q^{\left( {\begin{array}{c}n+1\\ 2\end{array}}\right) }(-a)^{-n}\Big (\frac{q}{a};q\Big )_n^{-1} \end{aligned}$$

(an identity which is actually valid for any $n\in \mathbb {Z}$). We will also use the common notational product convention

$$\begin{aligned}  &   (a_1, \ldots , a_k;q)_b:= (a_1;q)_b\cdots (a_k;q)_b, \end{aligned}$$

where $b\in {\mathbb {C}}\cup \{\infty \}$. The *theta function* $\vartheta (z;q)$ (sometimes referred to as a modified theta function [5, (11.2.1)]) is defined by Jacobi’s triple product identity and is given by [5, (1.6.1)] (see also [9, (2.3)])

5 $$\begin{aligned} \vartheta (z;q):= (z,\tfrac{q}{z};q)_\infty =\frac{1}{(q;q)_\infty }\sum _{n=-\infty }^\infty q^{\left( {\begin{array}{c}n\\ 2\end{array}}\right) }(-z)^n, \end{aligned}$$

where $z\ne 0$, $|q|<1$. Note that $\vartheta (q^n;q)=0$ if $n\in \mathbb {Z}$. We will use the compact product notation

$$\begin{aligned} \vartheta (z_1,\ldots ,z_k;q):=\vartheta (z_1;q)\cdots \vartheta (z_k;q). \end{aligned}$$

#### Lemma 1.2

One has the following modified theta function addition formula [5, Exercise 2.16(i)]

6 $$\begin{aligned} {\vartheta }\Big (e,\frac{e}{c},\frac{qa}{d},\frac{qc}{ad};q\Big ) -{\vartheta }\Big (d,\frac{d}{c},\frac{qa}{e},\frac{qc}{ae};q\Big )= \frac{d}{c}\,{\vartheta }\Big (a,\frac{c}{a},\frac{e}{d},\frac{de}{c};q\Big ). \end{aligned}$$

The *q*-shifted factorial also has the following useful property [8, (1.8.10)]:

7 $$\begin{aligned} (a;q^{-1})_n=q^{-\left( {\begin{array}{c}n\\ 2\end{array}}\right) }(-a)^n(a^{-1};q)_n. \end{aligned}$$

Note the equivalent representation of (7) which is very useful for obtaining limits of basic hypergeometric and bilateral basic hypergeometric series,

$$\begin{aligned} a^n\left( \frac{x}{a};q\right) _n= q^{\left( {\begin{array}{c}n\\ 2\end{array}}\right) }(-x)^n\left( \frac{a}{x};q^{-1}\right) _n, \end{aligned}$$

therefore

8 $$\begin{aligned} \lim _{a\rightarrow 0}\,a^n\left( \frac{x}{a};q\right) _n= \lim _{b\rightarrow \infty }\,\frac{1}{b^n}\left( xb;q\right) _n= q^{\left( {\begin{array}{c}n\\ 2\end{array}}\right) }(-x)^n. \end{aligned}$$

Furthermore, one has the following identities [2, (5), (6)],

9 $$\begin{aligned}  &   (a^2;q)_\infty =(\pm a,\pm q^\frac{1}{2} a;q)_\infty ,\end{aligned}$$

10 $$\begin{aligned}  &   \frac{(a,\frac{q}{a};q)_\infty }{(qa,\frac{1}{a};q)_\infty }= \frac{{\vartheta }(a;q)}{{\vartheta }(qa;q)} =-a. \end{aligned}$$

Finally, we will use the following *capital I* notation11($f(x_1,\ldots ,x_k)$ being any function depending on $x_1,\ldots ,x_k$), appearing e.g., in (38), (39). This means that the whole expression after the large *I* symbol on the left is taken as a summand and is then additively repeated, with $x_2$ switched with $x_1$, then with $x_3$ switched with $x_1$, etc., and finally with $x_k$ switched with $x_1$, the sum having *k* terms in total. We refer to this notation as *idem* notation (which we employ instead of the notation $\operatorname {idem} (x_1;x_2,\ldots ,x_k) f(x_1,\ldots ,x_k)$) frequently appearing in the literature on bilateral (basic) hypergeometric series, see e.g., Sears (1951) [14, p. 481], Slater (1966) [16, p. 192], [5, (4.5.2)]).

## Unilateral basic hypergeometric series transformations

For (ordinary) hypergeometric series (sometimes called *generalized hypergeometric series*, and denoted as $_rF_s$ series, to distinguish them from the classical hypergeometric $_2F_1$ function, extensively studied by Euler, Gauss, Riemann, Kummer, etc.), we refer the reader to the classic textbook by Slater [16].

The basic hypergeometric series (cf. [5]), which we frequently use, is defined for $q,z\in {{\mathbb {C}}^*}$ such that $|q|<1$, $s,r\in \mathbb {N}_0$, $b_j\not \in q^{-\mathbb {N}_0}$, $j=1,...,s$, as [8, (1.10.1)]12whenever the series converges. This nonterminating infinite series (12) is entire if and only if $s-r+1 > 0$, is convergent for $s=r-1$ if $|z|<1$ and is divergent for $s-r+1<0$. We sometimes specifically refer to the series in (12) as a *unilateral* basic hypergeometric series, to distinguish it from a *bilateral* basic hypergeometric series that we consider in Sect. 3.

Note that we refer to a basic hypergeometric series as *balanced* if $qa_1\cdots a_r=b_1\cdots b_s$. Further, define the nonterminating very-well-poised basic hypergeometric series ${}_{r+1}W_r$ [5, (2.1.11)]13where $\sqrt{a},\frac{qa}{a_4},\ldots ,\frac{qa}{a_{r+1}}\not \in q^{-\mathbb {N}_0}$. We will use the following notation $_{r\!}^{\phantom {\mid }}\mathop {{\mathop {\mathop {\phi }}\nolimits _{s}^{m}}}\limits$, $m\in \mathbb {N}_0$ (originally due to van de Bult and Rains [17, p. 4]), for basic hypergeometric series when some denominator elements are equal to zero. Let $p\in \mathbb {N}_0$. Then define, for multisets $\textbf{a}:=\{a_1,\ldots ,a_{r+1}\}$ and $\textbf{b}:=\{b_1,\ldots ,b_s\}$,1415where $b_1,\ldots ,b_s\not \in q^{-\mathbb {N}_0}\cup \{0\}$, and ${}_{r+1}\phi _s^0 ={}_{r+1}\!\phi _{s} .$ The nonterminating basic hypergeometric series $_{r+1}\mathop {{\mathop {\mathop {\phi }}\nolimits _{s}^{m}}}\limits (\textbf{a};\textbf{b};q,z)$ is well-defined for $s-r+m\ge 0$. In particular, ${}_{r+1}\phi _s^m$ is an entire function of *z* for $s-r+m>0$, convergent for $|z|<1$ for $s-r+m=0$ and divergent if $s-r+m<0$ unless the series is terminating. Note that we will move interchangeably between the van de Bult and Rains notation and the alternative notation with vanishing numerator and denominator elements which are used on the right-hand side of (15).

Further, consider the nonterminating very-well-poised basic hypergeometric series with vanishing denominator elements ${}_{r+1}\!W_r^p$ [17, p. 4]16where $\sqrt{a},\frac{qa}{a_4},\ldots ,\frac{qa}{a_{r+1}}\not \in q^{-\mathbb {N}_0}$. Recognize that ${}_{r+1}\!W_r^0={}_{r+1}\!W_r$. Note that in the case where $p<0$, then the nonterminating basic hypergeometric series ${}_{r+1}\!W_r^p$ is divergent since the corresponding nonterminating basic hypergeometric series ${}_r\phi _s$ (12) has $s-r+1<0$ (and thus, the series contains negative quadratic powers of *q*, responsible for its divergence). Therefore we only consider the nonterminating case ${}_{r+1}\!W_r^p$ with $p\ge 0$.

### Nonterminating transformations

One has the following nonterminating transformation between a ${}_2\phi _2$ and a ${}_2\phi _1$ (cf. [5, (III.4)])17valid for $|bz|<|c|$. More generally, we have (cf. [5, (III.9), (III.10)])1819valid for $|de|<|abc|$, $|e|<|a|$, and $|b|<1$. In this paper we will make use of the following three-term $_2\phi _1$ transformation [5, (III.32)]20valid for $\max (|z|,|\frac{qc}{abz}|)<1$. There is also a useful transformation of a ${}_2\phi _1$ expressed as a sum of two ${}_3\phi _2$’s with one vanishing denominator element [4, (17.9.3_5)]21valid for $|z|<1$. We will also rely upon the following three-term nonterminating transformation for a special ${}_3\phi _2$ [4, (17.9.13)]22where $|de|<|abc|$. One also has the important transformation due to Bailey which gives a nonterminating ${}_8\!W_7$ in terms of a sum of two nonterminating balanced ${}_4\phi _3$’s [4, (17.9.16)]23where $|q^2a^2|<|bcdef|$, with no vanishing denominator elements.

By taking advantage of Bailey’s transformation of a very-well-poised nonterminating ${}_8\!W_7$ (23), we can obtain some interesting single basic hypergeometric series transformation formulas. The following sequence of transformation formulas is implied by [17, Figure 2].

#### Theorem 2.1

Let $0<|q|<1$, $z,a,b,c,d,e\in {{\mathbb {C}}^*}$. Then2425262728whenever the series converge.

#### Proof

For (24), start with Bailey’s transformation of a nonterminating very-well-poised ${}_8\!W_7$ to a sum of two nonterminating balanced ${}_4\phi _3$’s (23) and take the limit $b\rightarrow \infty$ (or $c\rightarrow \infty$), replace $f\mapsto b$ (or $f\mapsto c$), and then apply the three-term nonterminating transformation for a ${}_3\phi _2$ (22). For (25), start with (24), take the limit as $e\rightarrow \infty$ (or $d\rightarrow \infty$) produces the ${}_2\phi _2$ representation and taking the limit as $c\rightarrow \infty$ (or $b\rightarrow \infty$) and then replacing variables accordingly so that *b*, *c*, *d* remains, produces the result. For (26), starting with (25): (i) taking the limit in the ${}_2\phi _2$ representation as $d\rightarrow \infty$ produces the ${}_1\!\phi _2$ representation; (ii) taking the limit as $b\rightarrow \infty$ (or $c\rightarrow \infty$) in the ${}_2\!\phi _2$ representation or taking the limit as $b\rightarrow \infty$ (or $d\rightarrow \infty$) in the ${}_2\phi _1$ representation produces the ${}_1\!\phi _1$ representation; (iii) taking the limit as $c\rightarrow \infty$ in the ${}_2\phi _1$ representation produces the ${}_2\!\phi _0^1$ representation. For (27), starting with (26): (i) taking the limit as $c\rightarrow \infty$ (or $b\rightarrow \infty$) in the ${}_1\phi _2$ representation, or taking the limit $b\rightarrow \infty$ in the ${}_1\phi _1$ representation produces the ${}_0\phi _1$ representation; (ii) taking the limit $c\rightarrow \infty$ in the ${}_1\phi _1$ representation produce, or taking the limit as $c\rightarrow \infty$ (or $b\rightarrow \infty$) in the ${}_2\phi _0^1$ representation produces the ${}_1\phi _0^1$ representation. For (28), starting with (27), taking the limit as $b\rightarrow \infty$ in either the ${}_0\phi _1$ or ${}_1\phi _0^1$ representations produces the first result. The second result in (28) is obtained by using [6, (7.6)]. This completes the proof. $\square$

#### Remark 2.2

The transformations of the special ${}_3\phi _2(a,b,c;d,e;q,de/(abc))$ which appears in (24) is alluded to in [17, p. 20]. Also for the nonterminating transformation of the general ${}_2\phi _1(a,b;c;q,z)$ in (25), see [17, p. 23]. See especially [17, Figure 2] for a summary of the entire hierarchy, which follows from their analysis of the $m=1$ limits of functions associated with vertices of various polytopes.

#### Remark 2.3

The function appearing in (28)[11, p. 57] is what Ismail and collaborators refer to as the *Ramanujan function*. This function appears in the Plancherel–Rotach asymptotics for the Al-Salam–Chihara polynomials, see Dai, Ismail & Wang (2020) [3]. The second representation of the Ramanujan function is given in Gessel & Stanton (1983) [6, (7.6)].

## Bilateral basic hypergeometric series

For (ordinary) bilateral hypergeometric series, denoted as $_rH_s$ series, we refer the reader to the classic textbook by Slater [16]. A bilateral basic hypergeometric series (cf. [5, Ch. 5]) is defined as29where $b_1,\ldots ,b_s\notin q^{-\mathbb {N}_0}$. This series converges for $s\ge r$ provided that $|b_1\cdots b_s|<|a_1\cdots a_rz|$, and also in the case when $s=r$, $|z|<1$. If $s>r$ and there are no vanishing numerator elements, then the bilateral series represents a holomorphic function on $z\in {\mathbb {C}}\setminus \{0\}$ (with essential singularity at $z=0$). Define the nonterminating *very-well-poised* bilateral basic hypergeometric series ${}_{r}\Psi _r$ [5, (2.1.11)]30where $\pm \sqrt{a},\frac{qa}{a_3},\ldots ,\frac{qa}{a_{r}}\not \in q^{-\mathbb {N}_0}$.

We will use the following notation $_{r}^{\phantom {\mid }}\mathop {{\mathop {\mathop {\psi }}\nolimits _{s}^{m}}}\limits$, $m\ge r-s$, (an extension of the notation of van de Bult–Rains [17, p. 4]), for bilateral basic hypergeometric series when some denominator elements are equal to zero. Let $p\in \mathbb {N}_0$. Then define31where $\textbf{a}:=\{a_1,\ldots ,a_r\}$, $\textbf{b}:=\{b_1,\ldots ,b_s\}$, $b_1,\ldots ,b_s\not \in q^{-\mathbb {N}_0}\cup \{0\}$, and $_{r}\mathop {{\mathop {\mathop {\psi }}\nolimits _{s}^{0}}}\limits ={}_{r}\psi _{s} .$ Note that the corresponding nonterminating bilateral basic hypergeometric series ${}_r\psi _s$ (29) with numerator elements equal to zero is divergent, since the condition $|b_1\cdots b_s|<|a_1\cdots a_r z|$ is impossible to satisfy. The nonterminating bilateral basic hypergeometric series $_{r}\mathop {{\mathop {\mathop {\psi }}\nolimits _{s}^{m}}}\limits (\textbf{a};\textbf{b};q,z)$, $\textbf{a}:=\{a_1,\ldots ,a_{r}\}$, $\textbf{b}:=\{b_1,\ldots ,b_s\}$, is well-defined for $s-r+m\ge 0$. Note that we will move interchangeably between the van de Bult–Rains notation and the alternative notation with vanishing numerator and denominator elements which are used on the right-hand side of (31).

Let $p\in \mathbb {N}_0$. Further, consider the nonterminating very-well-poised bilateral basic hypergeometric series with vanishing denominator elements ${}_{r}\Psi _r^p$ [17, p. 4]32where $\pm \sqrt{a},\frac{qa}{a_3},\ldots ,\frac{qa}{a_{r}}\not \in q^{-\mathbb {N}_0}$. Recognize that ${}_{r}\Psi _r^0={}_{r}\Psi _r$. Note that in the case where $p<0$, then the nonterminating bilateral basic very-well-poised hypergeometric series ${}_{r}\Psi _r^p$ is divergent since for the corresponding nonterminating bilateral basic hypergeometric series ${}_r\psi _s$ (29), the condition $|b_1\cdots b_s|<|a_1\cdots a_r z|$ is impossible to satisfy. Therefore, we only consider the nonterminating case ${}_{r}\Psi _r^p$ with $p\ge 0$. We now present some important properties of bilateral basic hypergeometric series.

### Bilateral basic hypergeometric summations

First there is Ramanujan’s ${}_1\!\psi _1$ summation [4, (17.8.2)]33where $\big |\frac{b}{a}\big |<|z|<1$. We further have Bailey’s bilateral ${}_6\psi _6$ summation [4, (17.8.7)]34where $|qa^2|<|bcde|$.

### Several known bilateral basic hypergeometric transformations

There are several important transformations of bilateral basic hypergeometric series which we will need. The first is (cf. [4, (17.10.1)])35valid for $\big |\frac{cd}{ab}\big |<|z|<1$ and $\big |\frac{cd}{ab}\big |<\big |\frac{c}{a}\big |<1$. The next is given in Rosengren [12, p. 366]36where $|qb|<|c|$, $|cd|<|abz|$, $|z|<1$. Another important transformation for bilateral basic hypergeometric series is Bailey’s ${}_6\Psi _6^2$ (sometimes referred as ${}_6\psi _8$) to ${}_2\psi _2$ transformation cf. [5, Exercise 5.11]37where $|qa|<|bc|$ and $|qa|<|de|$. This identity was used in [13], in conjunction with the Jacobi triple product identity (5), to deduce a number of bilateral identities of the Rogers–Ramanujan type. In this paper we would like to consider generalizations and repercussions of the important bilateral transformation (37). Note that combining (35) and (37) gives many transformations of the ${}_6\Psi _6^2$ (or ${}_6\psi _8$).

### Transformations for the ${}_{10}\Psi _{10}$

An important transformation for the very-well-poised ${}_{10}\Psi _{10}$ can be obtained by evaluating Slater’s [15] ${}_{2r}\psi _{2r}$ bilateral transformation for $r=5$ (see [5, (5.5.2)]). First define the multiset ${\textbf{b}}:=\{b,c,d,e,f,g,h,k\}$. Then one has38provided $|q^3a^4|<|bcdefghk|$, where $\lambda ,\mu ,\nu$ are free parameters. By setting $(\lambda ,\mu ,\nu )=(g,h,k)$ in (38), one obtains the more-frequently encountered transformation for the very-well-poised ${}_{10}\Psi _{10}$, namely (cf. [4, (17.10.6)], [5, (III.40)])39provided $|q^3a^4|<|bcdefghk|$. The above transformation can be seen to be a generalization of (41) below, obtained by setting $k=qa/h$. Slater’s very-well-poised ${}_{10}\Psi _{10}$ transformation formula can principally be utilized, by taking limits of the numerator parameters *b*, *c*, *d*, *e*, *f* to infinity, to obtain transformations for the ${}_9\Psi _9^1$, ${}_8\Psi _8^2$, ${}_7\Psi _7^3$, ${}_6\Psi _6^4$, ${}_5\Psi _5^5$ functions. However, these functions are not included in the present study.

#### Remark 3.1

It’s important to recognize that even though Slater’s very-well-poised transformations (38), (39) are four-term bilateral transformations, they are *not* bilateral extensions of Bailey’s four-term very-well-poised balanced ${}_{10}W_9$ transformations [5, (III.39) and Ex. 2.30] and also do *not* contain Bailey’s transformation of an $_8\!W_7$ series in terms of two balanced $_4\phi _3$ series [5, (III.36)] or in terms of other $_8\!W_7$ series [5, (III.37)].

### Transformations for the ${}_8\!\Psi _8$ series

In the following we turn to transformations involving ${}_8\!\Psi _8$ series. In addition to stating Slater’s transformation of ${}_8\!\Psi _8$ series in (40) (which is a special case of (38)), our focus is on rather recently obtained transformations of ${}_8\!\Psi _8$ series (by Zhang and Zhang [19], and by Wei and Yu [18]) that contain the Bailey transformations [5, (III.36)] and [5, (III.37)] mentioned in Remark 3.1. These will be reproduced in Theorems 3.3 and 3.13, together with some nice specializations, given as corollaries, that have not been explicitly stated before.

#### Remark 3.2

We would like to emphasize the difficulty in extending (and possibly even unifying) the results that are featured in Theorems 3.3 and 3.13 to the ${}_{10}\psi _{10}$ level. In particular, Zhang and Zhang [19] obtained their result by making use of Bailey’s nonterminating ${}_{10}W_9$ transformation and applying a bilateralization method (that necessarily reduces the order of the basic hypergeometric series), while Wei and Yu [18] used Ismail’s analytic continuation argument from [7] to lift a three-term transformation of $_8\!\phi _7$ series to one that involves two ${}_8\!\Psi _8$ series and one $_8\phi _7$ series. While Ismail’s argument has proven to be powerful in many cases, the argument can not be applied in the special setting of *balanced* series (such as in the series occurring in Bailey’s four-term ${}_{10}W_9$ transformation). While a bilateral series, summed over integers *j*, containing in its summands the factors $\frac{(\alpha z^{-1};q)_j}{(\beta z;q)_j}z^j$, can very well be analytic in the variable *z* (an argument used by Askey and Ismail [1] to extend Rogers’ $_6\phi _5$ summation [5, (II.20)] to Bailey’s $_6\psi _6$ summation (34)), a series over *j* containing the well-poised factors

$$ \frac{(\alpha z,\frac{\alpha }{z};q)_j}{(\beta z,\frac{\beta }{z};q)_j} $$

is *not* analytic in *z*. Thus, there is unfortunately no clear direct route to obtaining transformations for ${}_{10}\psi _{10}$ series that would contain the results given in Theorems 3.3 and 3.13.

An important transformation for the very-well-poised ${}_{8}\!\Psi _{8}$ can be obtained by evaluating [5, (5.5.2)] for $r=4$. Let $\lambda ,\mu ,a,b,c,d,e,f\in {{\mathbb {C}}^*}$ and define the multiset ${\textbf{b}}:=\{b,c,d,e,f,g\}$. Then one has the following transformation of an ${}_8\!\Psi _8$ as a symmetric sum of two very-well-poised ${}_8\!\Psi _8$’s, namely40provided $|q^2a^3|<|bcdefg|$, where $\lambda ,\mu$ are free parameters. By setting $(\lambda ,\mu )=(f,g)$ in (40), one obtains the more-frequently encountered transformation for the very-well-poised ${}_{8}\!\Psi _{8}$, namely [4, (17.10.5)], [5, (III.38)], a symmetric sum of two very-well-poised ${}_8\!W_7$’s, namely41Note that the transformations (40), (41) are invariant under parameter interchange of the six variables *b*, *c*, *d*, *e*, *f*, *g*. For a direct proof of (41), see [5, §5.6].

As mentioned in Sect. 2.1, we would like to consider generalizations of (37). In order to accomplish this, we now examine Zhang and Zhang’s transformation [19, Theorem 6] which is is a transformation for a very-well-poised ${}_8\!\Psi _8$ series that constitutes a quite notable generalization of Bailey’s transformation (23). However, the result of Zhang–Zhang is incorrectly stated in their paper; we provide a correction to it, and provide it in a form such that the $_8\!\Psi _8$ series is manifestly symmetric in six of its parameters, which will be the contents of Theorem 3.3.

First we correct the Zhang–Zhang result [19, Theorem 6]. Specifically, the first balanced $_4\phi _3$ in their formula must be replaced by the ${}_4\,\phi _3(b,c,d,e;\frac{qa}{f},\frac{qa}{g},\frac{qa}{h};q,q)$ series, which gives42where $\lambda =\frac{qa^2}{cde}$, $h=\frac{q^2a^3}{bcdefg}$. Now, with this correction, writing out $\lambda$ and *h* by their defining relations, and making the replacement

$$ \{a,b,c,d,e,f,g\}\mapsto \left\{ \frac{qa^2}{bcd},\frac{q^2a^3}{bcdefg},\frac{qa}{cd},\frac{qa}{bd},\frac{qa}{bc},e,f\right\} , $$

the corrected result can be restated (now with the very-well-poised ${}_8\psi _8$ series on one side alone) as follows.

#### Theorem 3.3

Zhang and Zhang (2007) [19, Theorem 6]. Let $0<|q|<1$, $a,b,c,d,e,f,g\in {{\mathbb {C}}^*}$ such that $|q^2a^3|<|bcdefg|$ such that none of the denominator factors vanish. Then one has the following transformation formula for a very-well-poised ${}_8\Psi _8$, namely43

#### Remark 3.4

Letting $(g,d)\mapsto (q^{-n},q^{-n}a)$ in Theorem 3.3 followed by replacing $f\mapsto d$, reproduces [4, (17.10.3)], which, as a transformation involving terminating series on both sides, is equivalent to Watson’s transformation [5, (III.18)]. Instead, by only letting $g\mapsto q^{-n}$ (or $d\mapsto q^{-n}a$) in (42), an identity equivalent to Bailey’s transformation in (23) is obtained.

If, in (42), one substitutes $(a,b,c,d,e,f,g)\mapsto (q^a,q^b,q^c,q^d,q^e,q^f,q^g)$ and uses the definition of the *q*-gamma function, then one obtains the following transformation for very-well-poised bilateral ${}_7H_7$ to a ${}_4H_4$ and two nonterminating balanced ${}_4F_3$’s, all series with argument unity.

#### Theorem 3.5

Let $$a,b,c,d,e,f,g\in {{\mathbb {C}}^*}$$. Then44provided $$\Re (2+3a-b-c-d-e-f-g)>0$$.

#### Proof

Starting with the Zhang–Zhang transformation in Theorem 3.3, we substitute

$$ (a,b,c,d,e,f,g)\mapsto (q^a,q^b,q^c,q^d,q^e,q^f,q^g) $$

and rewrite ratios of infinite *q*-shifted factorials using the *q*-gamma function, defined by [4, (5.18.4)]

45 $$\begin{aligned} \Gamma _q(z):=\frac{(q;q)_\infty (1-q)^{1-z}}{(q^z;q)_\infty }. \end{aligned}$$

Then we take the limit as $q\rightarrow 1^{-}$ on both sides^1^, using $\lim _{q\rightarrow 1^{-}}\Gamma _q(z)=\Gamma (z)$ and applying [8, (1.8.4)]

46 $$\begin{aligned} \lim _{q\rightarrow 1^{-}}\frac{(q^\alpha ;q)_k}{(1-q)^k}=(\alpha )_k, \end{aligned}$$

where $(\alpha )_k:=(\alpha )(\alpha +1)\cdots (\alpha +k-1)$ is a rising factorial. The expression is thus reduced from the respective bilateral and unilateral basic hypergeometric functions to corresponding ordinary hypergeometric functions, completing the proof (where we have omitted the technical details pertaining to rigourously justifying the term-wise $q\rightarrow 1^{-}$ limit). $\square$

#### Remark 3.6

Note that one can obtain similar transformations of bilateral hypergeometric series by taking the limit as $q\rightarrow 1^{-}$ of transformations which are derived below. However, the details are routine and are omitted.

#### Remark 3.7

As noticed in [19, Corollary 8], setting $c=a$ in Theorem 3.3 and replacing $g\mapsto c$ produces Bailey’s transformation of a nonterminating ${}_8\!W_7$ in terms of a sum of two balanced nonterminating $_4\phi _3$’s with argument *q* (23).

The transformation in Theorem 3.3 involves series that are manifestly symmetric in *b*, *c*, *d* and, separately, in *e*, *f*, *g*. Using the simple identity $(z,\omega z,\omega ^2 z;q)_k=(z^3;q^3)_k$, where $\omega$ is a fixed primitive third root of unity, we are able to derive the following cubic summation formula involving a bilateral series:

#### Corollary 3.8

Let $0<|q|<1$ and $a,b\in {{\mathbb {C}}^*}$. Then47where $\omega$ is a fixed primitive third root of unity.

#### Proof

We sketch the details. In (43), we make the substitutions

$$\begin{aligned} (b,c,d,e,f,g)\mapsto (b^{\frac{1}{3}},\omega b^{\frac{1}{3}},\omega ^2 b^{\frac{1}{3}}, q^{\frac{1}{3}}ab^{-\frac{1}{3}},\omega q^{\frac{1}{3}}ab^{-\frac{1}{3}}, \omega ^2 q^{\frac{1}{3}}ab^{-\frac{1}{3}}), \end{aligned}$$

where $\omega$ is a fixed primitive third root of unity, and where for $q^{\frac{1}{3}}$ and $b^{\frac{1}{3}}$ fixed branches of the respective third roots are taken. Under these substitutions, the argument of the $_8\Psi _8$ series becomes *q*, the $_4\Psi _4$ reduces to a $_1\Psi _1$ series of base $q^3$, and the two $_4\phi _3$ series reduce to Fine–type basic hypergeometric series with base $q^3$ (i.e., to series of the form $_2\phi _1(a,q^3;c;q^3,z)$ which can also be viewed as the half of a bilateral $_1\Psi _1$ series). The $_1\Psi _1$ series can be simplified by virtue of the $(q,a,b,z)\mapsto (q^3,\frac{qa^3}{b},\frac{q^3a^3}{b},q)$ case of Ramanujan’s $_1\Psi _1$ summation formula (33), while to the first $_2\phi _1$ series we apply the $(q,a,b,c,z)\mapsto (q^3,\frac{q^3a^3}{b^2},q^3,\frac{q^5a^3}{b^2},q)$ case of (20). Simplification yields the claimed result. $\square$

By letting $b=a^3$ in Corollary 3.8 we immediately obtain the following simpler identity involving unilateral series:

#### Corollary 3.9

Let $0<|q|<1$ and $a\in {{\mathbb {C}}^*}$. Then48where $\omega$ is a fixed primitive third root of unity.

#### Remark 3.10

One can obtain a closed summation from Corollary 3.8 by specializing the parameters such that the ${}_2\phi _1$ reduces to its first term. This is achieved by letting $qb^2=a^3$. In this case, several of the terms in (47) vanish and on the right-hand side one is left with only one term (and in addition, several of the factors cancel). However, the identity thus obtained is nothing new but just the $(a,b,c,d,e,q)\mapsto (a,q^{-\frac{1}{2}}a^{\frac{3}{2}},\omega q^{-\frac{1}{2}}a^{\frac{3}{2}}, \omega ^2 q^{-\frac{1}{2}}a^{\frac{3}{2}},q^{\frac{1}{2}}a^{\frac{1}{2}},q)$ special case of Bailey’s very-well-poised $_6\psi _6$ summation (34).

#### Remark 3.11

Since a cubic specialization of Theorem 3.3 yielded a new result, one might be tempted to try a quartic specialization that makes use of the simple identity $(z, -z, \mathrm iz,-\mathrm iz;q)_k=(z^4;q^4)_k$, where $\mathrm i$ is the imaginary unit. The purpose of such a specialization may be to reduce the $_4\psi _4$ in (43) to a summable $_1\psi _1$ (by Ramanujan’s formula in (33)) with base $q^4$. This works by replacing (*a*, *b*, *c*, *d*, *e*, *f*, *g*, *q*) by $(a,b,\mathrm ib,-\mathrm ib,qa^2b^{-3},\mathrm iqa^2b^{-3},-\mathrm iqa^2b^{-3},q)$ but at the same time reduces the two $_4\phi _3$ series in (43) to summable $_1\phi _0$ series. As a result, a summation for a bilateral series is obtained into a sum of three infinite *q*-products. However, closer inspection of the thus obtained identity reveals that the three terms of infinite *q*-products can be reduced, by two applications of the theta function addition formula (6), to a single term, and the bilateral series that is left is just a specialized $_4\psi _4$ series of base $q^4$. It turns out that the identity obtained in the described manner is actually just a special case of the well-known bilateral analogue of the *q*-Dixon sum in [5, (II.32)].

A principal interesting special case of (43) corresponds to the case of setting *g* to *a*. In this case the ${}_8\Psi _8$ becomes an ${}_8W_7$ and we produce the following result.

#### Corollary 3.12

Let $0<|q|<1$, $a,b,c,d,e,f\in {{\mathbb {C}}^*}$ such that $|q^2a^2|<|bcdef|$. Then49

#### Proof

Start with (43) and setting $e=a$ converts the ${}_8\Psi _8$ to an ${}_8W_7$. Then replace $g\mapsto e$ and simplification completes the proof. $\square$

Next we recall an interesting transformation formula first derived by Wei and Yu [18, Theorem 3] for a very-well-poised ${}_8\Psi _8$. This transformation formula provides a generalization of the two and three-term transformations for an ${}_8W_7$ [5, (III.23), (III.37)]. Note that in the following transformation, while the left-hand side is manifestly symmetric in the six variables *b*, *c*, *d*, *e*, *f*, *g*, the right-hand side satisfies the same symmetry (which otherwise is not obvious).

#### Theorem 3.13

Wei and Yu (2019) [18, Theorem 3]. Let $0<|q|<1$, $a,b,c,d,e,f,g\in {{\mathbb {C}}^*}$ such that $|q^2a^3|<|bcdefg|$, $|qa|<|fg|$, and none of the denominator factors vanish. Then one has the following transformation formula for a very-well-poised ${}_8\Psi _8$, namely50

#### Remark 3.14

Wei and Yu noticed [18, p. 3], if one sets $e=qa/d$ in Theorem 3.13, the left-hand side becomes a ${}_6\Psi _6$, the first term on the right-hand side vanishes, and the ${}_8W_7$ becomes a nonterminating ${}_6W_5$ which can then be summed using [4, (17.7.15)], and the identity reduces to Bailey’s ${}_6\Psi _6$ summation [4, (17.8.7)].

#### Remark 3.15

The bilateral basic hypergeometric transformation (50) is seen to be a generalization of the two-term transformation [5, (III.23)] by setting $b=a$ and then utilizing [5, Exercise 2.16]. It is also a generalization of the three-term transformation [5, (III.37)], obtained by setting one of *c*, *d*, *e*, *f*, *g* to *a*.

By setting $g=a/b$ in Theorem 3.13, then one obtains the following nonterminating summation formula involving two ${}_8\Psi _8$.

#### Corollary 3.16

Let $0<|q|<1$, $a,b,c,d,e,f\in {{\mathbb {C}}^*}$ such that $|q^2a^2|<|cdef|$, $|qb|<|f|$, and none of the denominator factors vanish. Then one has the following summation formula for an ${}_8\Psi _8$, namely

51 $$\begin{aligned}  &   \,_{8}\Psi _{8}\!\left( {a};{b,c,d,e,f,\frac{a}{b}};{q,\frac{q^2a^2}{cdef}}\right) \nonumber \\  &   \quad = \frac{( qa,\frac{q}{a},\frac{b}{a},\frac{qb}{f},\frac{qa}{cd},\frac{qa}{ce},\frac{qa}{de},\frac{bcd}{a},\frac{bce}{a}, \frac{bde}{a},\frac{q^2a}{cde},\frac{q^2a^2}{bcdef};q)_\infty }{(\frac{q}{f},\frac{qb}{a},\frac{qa}{c},\frac{qa}{d},\frac{qa}{e},\frac{bc}{a},\frac{bd}{a},\frac{be}{a},\frac{qa}{cde},\frac{bcde}{a},\frac{q^2a}{bcde},\frac{q^2a^2}{cdef};q)_\infty } \,_{8}\Psi _{8}\!\left( {\frac{bcde}{qa}};{b,c,d,e,\frac{bcdef}{qa^2},\frac{cde}{qa}};{q,\frac{qb}{f}}\right) \nonumber \\  &   \qquad +\frac{(q,q,qa,\frac{q}{a},c,d,e,\frac{qb}{c},\frac{qb}{d},\frac{qb}{e},\frac{qb}{f},\frac{qa}{bf};q)_\infty {\vartheta }(\frac{bcde}{qa^2};q)}{(qb,\frac{q}{b},\frac{q}{f},\frac{qb}{a},\frac{qa}{b},\frac{qa}{c},\frac{qa}{d},\frac{qa}{e},\frac{qa}{f},\frac{bc}{a},\frac{bd}{a},\frac{be}{a};q)_\infty {\vartheta }(\frac{cde}{qa};q)}. \end{aligned}$$

#### Remark 3.17

By taking $f=a$ in (51) we immediately obtain the summation formula

52 $$\begin{aligned} \,_{8}W_{7}\!\left( {a};{b,c,d,e,\frac{a}{b}};{q,\frac{q^2a}{cde}}\right)= &   \frac{( qa,\frac{b}{a},\frac{qa}{cd},\frac{qa}{ce},\frac{qa}{de},\frac{bcd}{a},\frac{bce}{a}, \frac{bde}{a};q)_\infty }{(\frac{qa}{c},\frac{qa}{d},\frac{qa}{e},\frac{bc}{a},\frac{bd}{a},\frac{be}{a},\frac{qa}{cde},\frac{bcde}{a};q)_\infty } \nonumber \\    &   \times \,_{8}W_{7}\!\left( {\frac{bcde}{qa}};{b,c,d,e,\frac{cde}{qa}};{q,\frac{qb}{a}}\right) \nonumber \\  &   +\frac{(q,qa,c,d,e,\frac{qb}{c},\frac{qb}{d},\frac{qb}{e};q)_\infty {\vartheta }(\frac{bcde}{qa^2};q)}{(qb,\frac{qa}{b},\frac{qa}{c},\frac{qa}{d},\frac{qa}{e},\frac{bc}{a},\frac{bd}{a},\frac{be}{a};q)_\infty {\vartheta }(\frac{cde}{qa};q)}, \end{aligned}$$

valid for $0<|q|<1$, $a,b,c,d,e\in {{\mathbb {C}}^*}$ such that $|q^2a|<|cde|$, $|qb|<|a|$ and none of the denominator factors vanish. This summation can also be obtained from specializing [5, (2.11.1)] by taking $bc=a$ (which reduces the third ${}_8W_7$ series to 1), application of the transformation of nonterminating $_8\!W_7$ series in [5, (2.10.1)] to the second ${}_8\!W_7$ series, and relabeling.

One can also obtain a nonterminating summation formula involving two ${}_8\!\Psi _8$ by starting with Theorem 3.13 and utilizing the nonterminating very-well-poised ${}_8\!W_7$ summation [5, (II.16)]

53 $$\begin{aligned} \,_{8}W_{7}\!\left( {\sqrt{\frac{ab}{q}}c};{a,b,\pm c,\frac{\sqrt{qab}}{c}};{q,-\frac{\sqrt{q}c}{\sqrt{ab}}}\right) =\frac{(\sqrt{qab}c, \frac{\sqrt{q}c}{\sqrt{ab}};q)_\infty (qa,qb,\frac{qc^2}{a},\frac{qc^2}{b};q^2)_\infty }{(\sqrt{\frac{qa}{b}}c,\sqrt{\frac{qb}{a}}c;q)_\infty (q,qab,qc^2,\frac{qc^2}{ab};q^2)_\infty }. \end{aligned}$$

#### Corollary 3.18

Let $0<|q|<1$, $a,b,c,d\in {{\mathbb {C}}^*}$ such that $|qa|<|cd|$, $|q^{1/2}b|<|\sqrt{cd}|$ and none of the denominator factors vanish. Then one has the following summation formula for the sum of two ${}_8\Psi _8$’s, namely

54 $$\begin{aligned}  &   \hspace{-0.5cm}\,_{8}\Psi _{8}\!\left( {a};{b,c,d,\pm \frac{\sqrt{q}a}{\sqrt{cd}},\frac{cd}{b}};{q,-\frac{qa}{cd}}\right) \nonumber \\  &   \hspace{0.0cm}= \frac{( qa,\frac{q}{a},-\frac{q}{b},\frac{b}{a},-\sqrt{\frac{qc}{d}},-\sqrt{\frac{qd}{c}},\frac{qa}{cd},\frac{\sqrt{q}b}{\sqrt{cd}},\frac{bcd}{a},-\sqrt{\frac{qc}{d}}b,-\sqrt{\frac{q2d}{c}}b,-\frac{q^{3/2}a}{(cd)^{3/2}};q)_\infty }{(-\sqrt{qcd},-\sqrt{qcd}b,\frac{qa}{c},\frac{qa}{d},\frac{bc}{a},\frac{bd}{a},\frac{qb}{cd},\frac{\sqrt{qcd}}{a},-\frac{\sqrt{q}}{\sqrt{cd}},-\frac{\sqrt{q}b}{\sqrt{cd}},-\frac{q^{3/2}}{\sqrt{cd}b},-\frac{qa}{cd};q)_\infty }\nonumber \\  &   \hspace{0.7cm}\quad \times \,_{8}\Psi _{8}\!\left( {-\frac{\sqrt{cd}b}{\sqrt{q}} };{\pm b,c,d,-\frac{\sqrt{q}a}{\sqrt{cd}},-\frac{(cd)^{3/2}}{\sqrt{q}a}};{q,\frac{\sqrt{q}b}{\sqrt{cd}}}\right) \nonumber \\  &   \hspace{0.7cm}+\frac{(q,qa,\frac{q}{a},c,d,\frac{qa}{cd},\frac{qa}{cd},-\frac{\sqrt{q}a}{\sqrt{cd}},\frac{\sqrt{qcd}}{b},\frac{qb^2}{cd};q)_\infty {\vartheta }(-\sqrt{\frac{cd}{q}}\frac{b}{a};q)(\frac{qbc}{a},\frac{qbd}{a},\frac{q^2ab}{c^2d},\frac{q^2ab}{cd^2};q^2)_\infty }{(\frac{q}{b},\frac{qa}{b},\frac{qa}{c},\frac{qa}{d},\frac{bc}{a},\frac{bd}{a},\frac{\sqrt{qcd}}{a},\frac{qb}{cd},-\frac{\sqrt{q}b}{\sqrt{cd}},\frac{qab}{cd};q)_\infty {\vartheta }(-\sqrt{\frac{cd}{q}};q)(q,qcd,\frac{q^2b^2}{cd},\frac{q^2a^2}{c^2d^2};q^2)_\infty }. \nonumber \\ \end{aligned}$$

#### Proof

Start with Theorem 3.13 and replace

$$ (g,f,e)\mapsto \left( - \frac{\sqrt{qcd}a}{be} ,-e,- \frac{\sqrt{q}a}{\sqrt{cd}}\right) . $$

Then the resulting very-well-poised ${}_8W_7$ can be summed using [5, (II.16)]. Simplifying the final result completes the proof. $\square$

One can also obtain a nonterminating summation formula involving two ${}_8\Psi _8$ by starting with Theorem 3.13 and utilizing the nonterminating very-well-poised ${}_8W_7$ summation [5, (II.18)]

55 $$\begin{aligned}  &   \hspace{1.7cm}\,_{8}W_{7}\!\left( {a};{-a,b,\frac{q}{b},c, \frac{q}{c}};{q,-a}\right) =\frac{(a,qa;q)_\infty (abc,\frac{qab}{c},\frac{qac}{b},\frac{q^2a}{bc};q^2)_\infty }{(ab,ac,\frac{qa}{b},\frac{qa}{c};q)_\infty }. \end{aligned}$$

#### Corollary 3.19

Let $0<|q|<1$, $a,b,c,d\in {{\mathbb {C}}^*}$ such that $|b^2|<|a|$, and none of the denominator factors vanish. Then one has the following summation formula for the sum of two ${}_8\Psi _8$’s related by the substitution $(a,d)\mapsto (-a,-d)$, namely

56 $$\begin{aligned}  &   \,_{8}\Psi _{8}\!\left( {a};{\pm b,c,d,\frac{qa^2}{b^2c},\frac{qa^2}{b^2d}};{q,-\frac{b^2}{a}}\right) \nonumber \\  &   \quad = \frac{( qa,\frac{q}{a},\frac{b}{a},-\frac{q}{d},\frac{b^2}{a},\frac{qa}{b},-\frac{qa}{c},-\frac{bc}{a},-\frac{b^2c}{a},-\frac{qa}{bc}, -\frac{b^2d}{a^2};q)_\infty }{(-qa,-\frac{q}{a},\frac{q}{d},-\frac{b}{a},-\frac{qa}{b},\frac{qa}{c},\frac{bc}{a},-\frac{b^2}{a},-\frac{b^2}{a},\frac{qa}{bc},\frac{b^2c}{a},\frac{b^2d}{a^2};q)_\infty } \nonumber \\    &   \qquad \quad \times \,_{8}\Psi _{8}\!\left( {-a};{\pm b,c,-d,\frac{qa^2}{b^2c},-\frac{qa^2}{b^2d}};{q,\frac{b^2}{a}}\right) \nonumber \\  &   \qquad +\frac{(qa,-b,c,\frac{q}{a},\frac{b^2}{a},\frac{bd}{a},\frac{qa}{bd},\frac{qa^2}{b^2c};q)_\infty (q^2,\frac{q^2a}{cd},\frac{b^4cd}{a^3},\frac{qb^2c}{ad},\frac{qb^2d}{ac};q^2)_\infty {\vartheta }(-1;q)}{(\frac{q}{b},\frac{q}{d},-\frac{b^2}{a},\pm \frac{qa}{b},\frac{qa}{c},\frac{qa}{d},\frac{bc}{a},\frac{b^2c}{a},\frac{b^2d}{a},\frac{b^2d}{a},\frac{b^2d}{a^2},\frac{qa}{bc};q)_\infty {\vartheta }(-\frac{a}{b};q)}. \end{aligned}$$

#### Proof

Start with Theorem 3.13 and replace

$$ (d,e,g)\mapsto \left( \frac{qa^2}{b^2c},-b,\frac{qa^2}{b^2f}\right) . $$

Then the resulting very-well-poised ${}_8W_7$ can be summed using [5, (II.18)]. Finally replacing $f\mapsto d$ and simplifying the final result completes the proof. $\square$

#### Remark 3.20

By taking $b=a$ in Corollaries 3.18 and 3.19, respectively, it is not difficult to see that these are bilateral extensions of the nonterminating very-well-poised ${}_8W_7$ summations [5, (II.16) and (II.18)] (53) and (55), respectively.

By equating the Zhang–Zhang and Wei–Yu transformations we can obtain a new transformation for a very-well-poised ${}_8\Psi _8$ series in terms of a ${}_4\psi _4$, an ${}_8W_7$ and two balanced ${}_4\phi _3$ series.

#### Corollary 3.21

Let $0<|q|<1$, $a,b,c,d,e,f,g\in {{\mathbb {C}}^*}$ such that $|q^2a^3|<|bcdefg|$, $|qa|<|fg|$, and such that none of the denominator factors vanish. Then one has the following transformation formula for a very-well-poised ${}_8\Psi _8$, namely57

#### Proof

First equate (43) and (50), then making the replacements

$$ (a,f,g)\mapsto \left( \frac{bcde}{qa},\frac{qa^2f}{bcde},\frac{qa^2\,g}{bcde}\right) , $$

and solving for the ${}_8\Psi _8$ completes the proof. $\square$

#### Remark 3.22

The $b\rightarrow a$ limit of Corollary 3.21 is equivalent to Gasper and Rahman [5, (III.23)].

## Very-well-poised bilateral transformations as limiting cases

In this section we consider transformations of ${}_{8-p}\Psi _{8-p}^p$ series, for $1\le p\le 6$, obtained as limiting cases of transformations for the ${}_8\Psi _8$ series presented above. First we consider the case $p=1$, namely transformations for the ${}_7\Psi _7^1$ series.

### Transformations for the ${}_7\Psi _7^1$ series

Starting with (41) leads to a nice corollary as a transformation of a very-well-poised ${}_7\Psi _7^1$. Note that in the following transformation, while the left-hand side is manifestly symmetric in the five variables *b*, *c*, *d*, *e*, *f*, the right-hand side satisfies the same symmetry (which otherwise is not obvious).

#### Corollary 4.1

Let $0<|q|<1$, $a,b,c,d,e,f\in {{\mathbb {C}}^*}$, $|q|<\max \{|de/a|,|df/a|,|cd/a|\}<1$. Then one has the following transformation for a very-well-poised ${}_7\Psi _7^1$ series58596061

#### Proof

Start with (41) and take the limit as $e\rightarrow \infty$. This converts the very-well-poised ${}_8\!\Psi _8$ to the very-well-poised ${}_7\Psi _7^1$ given on the left-hand side of (58). Taking the limit of both ${}_8\!W_7$’s on the right-hand side of (41) as $e\rightarrow \infty$ converts both ${}_8\!W_7$’s to ${}_7\!W_7$’s with one vanishing denominator element. These can be converted to ${}_3\phi _2$’s using the transformation (24), establishing (58). Starting with (43) instead and letting $b\mapsto q^{-n}a$ (or $c\mapsto q^{-n}a$), the coefficient of the second series on the right-hand side vanishes. After taking the limit $n\rightarrow \infty$, the identity becomes (59) up to a simple substitution of variables. To get (60) start with (50), let $g\rightarrow \infty$ and apply (24). Finally, we sketch how to get (61): Apply (58) to (60) to obtain a transformation of a very-well-poised ${}_7\Psi _7^1$ series into a sum of three ${}_3\phi _2$ series of which two ${}_3\phi _2$ series are the same but have different prefactors. The two different prefactors of the same ${}_3\phi _2$ series can be combined by virtue of the addition formula for the modified theta function in (6). The third ${}_3\phi _2$ series can finally be transformed using (18), completing the sketch of proof of (61) (while leaving the full details to the reader). $\square$

#### Corollary 4.2

Let $0<|q|<1$, $a,b,c,d,e,f\in {{\mathbb {C}}^*}$, $|q|<\max \{|de/a|,|df/a|,|cd/a|\}<1$. Then one has the following transformation for a very-well-poised ${}_7\Psi _7^1$62

#### Proof

This result is obtained by starting with (43) and taking the limit as $g\rightarrow \infty$. The final term vanishes and this completes the proof. $\square$

By starting with Corollary 3.21 and taking the limit as $g\rightarrow \infty$ one obtains a new transformation for a ${}_7\Psi _7^1$ in terms of a ${}_3\psi _3$ and two ${}_3\phi _2$’s.

#### Corollary 4.3

Let $0<|q|<1$, $a,b,c,d,e,f\in {{\mathbb {C}}^*}$, $|qa|<|ef|$. Then one has the following transformation for a very-well-poised ${}_7\Psi _7^1$ series:63

#### Proof

Start with Corollary 3.21 and take the limit as $g\rightarrow \infty$. In the limit the last term on the right-hand side of (57) vanishes. The ${}_4\psi _4$ becomes the ${}_3\psi _3$ and the ${}_8\!W_7$ becomes a ${}_7\!W_6^1$ which can be written as a ${}_3\phi _2$ using (24). This completes the proof. $\square$

### Transformations for the ${}_6\Psi _6^2$ series

Note that in the following transformation, while the left-hand side is manifestly symmetric in the four variables *b*, *c*, *d*, *e*, the right-hand side satisfies the same symmetry (which otherwise is not obvious).

#### Corollary 4.4

Let $0<|q|<1$, $b,c,d,e\in {{\mathbb {C}}^*}$. Then6465666768provided the series converge.

#### Proof of Corollary 4.4

Start with (58) and take the limit as $d\rightarrow \infty$, then replacing $f\mapsto d$ converts the ${}_3\phi _2$’s to ${}_2\phi _2$’s. Then one can use (17) twice to write the ${}_2\phi _2$’s in terms of ${}_2\phi _1$’s, hereby establishing (64). By taking the limit as $c\rightarrow \infty$ of (59) followed by $f\mapsto c$, we obtain a bilateral extension of (21) (after taking $c=a$), establishing (65). One can use (37) to write the left-hand side as a ${}_2\psi _2$, establishing (66). Equation (67) can be obtained by a direct limit from (60), while Equation (68) is obtained by combining (67) with (66). This completes the proof. $\square$

By starting with Corollary 4.3 and taking the limit as $f\rightarrow \infty$ one obtains a new transformation for a ${}_6\Psi _6^2$ in terms of a ${}_2\psi _3$, one ${}_2\phi _1$ and a ${}_3\phi _1^1$.

#### Corollary 4.5

Let $0<|q|<1$, $a,b,c,d,e,f\in {{\mathbb {C}}^*}$, $|q|<\max \{|de/a|,|df/a|,|cd/a|\}<1$. Then one has the following transformation for a very-well-poised ${}_6\Psi _6^2$69

#### Proof

Start with Corollary 4.3 and take the limit as $f\rightarrow \infty$. The ${}_3\psi _3$ becomes the ${}_2\psi _3$ and the first ${}_3\phi _2$ becomes a ${}_2\phi _2$ which can be written as a ${}_2\phi _1$ using (17). The last ${}_3\phi _2$ becomes a ${}_3\phi _1^1$ which completes the proof. $\square$

### Transformations for the ${}_5\Psi _5^3$ series

Note that in the following transformation, while the left-hand side is manifestly symmetric in the three variables *b*, *c*, *d*, the right-hand side satisfies the same symmetry (which otherwise is not obvious).

#### Corollary 4.6

Let $$0<|q|<1$$, $$a,b,c,d\in {{\mathbb {C}}^*}$$. Then70

#### Proof

Start with Corollary 4.4. Taking the limit as $e\rightarrow \infty$ of the left-hand side produces the left-hand side of (70). Taking the limit of the ${}_2\psi _1^2$ representation as $b\rightarrow \infty$ produces the ${}_1\psi _2$ representation. One can obtain the ${}_2\psi _1^1$ representation from the ${}_1\psi _2$ representation by reversing the order of summation. Taking the limit of the sum of the ${}_3\psi _1^2$ and ${}_3\phi _2$ representation as $\{e,c\}\rightarrow \infty$ produces the ${}_2\psi _1^1$ and ${}_3\psi _1^2$ plus ${}_3\phi _0^2$ representations respectively. This completes the proof. $\square$

### Transformations for the ${}_4\Psi _4^4$ series

Note that in the following transformation, while the left-hand side is manifestly symmetric in the two variables *b*, *c*, the right-hand side satisfies the same symmetry (which otherwise is not obvious).

#### Corollary 4.7

Let $$0<|q|<1$$, $$a,b,c\in {{\mathbb {C}}^*}$$. Then71

#### Proof

Start with Corollary 4.6. Taking the limit as $d\rightarrow \infty$ produces the left-hand side of (71). Taking the limit as $c\rightarrow \infty$ in the ${}_1\psi _2$ representation and replacing $d\mapsto c$ produces the ${}_1\!\psi _2$ representation. Taking the limit as $d\rightarrow \infty$ in the ${}_1\!\psi _2$ representation produces the ${}_0\psi _2$ representation. Taking the limit as $c\rightarrow \infty$ in the ${}_2\psi _2$ representation and replacing $d\mapsto c$ produces the ${}_2\psi _2$ representation. $\square$

### Transformations for the ${}_3\Psi _3^5$ series

#### Corollary 4.8

Let $$0<|q|<1$$, $$a,b\in {{\mathbb {C}}^*}$$. Then72

#### Proof

Start with Corollary 4.7. Taking the limit as $c\rightarrow \infty$ produces the left-hand side of (72). Taking the limit as $c\rightarrow \infty$ of the ${}_1\psi _2$ representation produces the ${}_1\psi _2$ representation. Taking the limit as $b\rightarrow \infty$ of the ${}_1\psi _2$ representation produces the ${}_0\psi _2$ representation. $\square$

### Summation for the ${}_2\Psi _2^6$ series

The following summation is a restatement of Watson’s quintuple product identity [4, (17.8.3)] (see also [5, Exercise 5.6]).

#### Corollary 4.9

Let $$0<|q|<1$$, $$a\in {{\mathbb {C}}^*}$$, $$a\ne 1$$. Then73

#### Proof

Start with Corollary 4.8, where each series is multiplied by $(1-a)$. Taking the limit as $b\rightarrow \infty$ of the ${}_1\psi _2$ or ${}_0\psi _2$ representations produces the ${}_0\psi _2$ representation which is evaluated using the triple-product identity (5). $\square$

## Implied bilateral basic transformations and summations

In this section we list some implications of the above transformations for very-well-poised bilateral basic hypergeometric series in terms of bilateral basic hypergeometric series that in general are not very-well-poised.

### Transformations for the balanced ${}_4\psi _4$ series

Using Corollary 3.21, one may obtain a transformation of a balanced ${}_4\psi _4$ in terms a two ${}_8\!W_7$’s and two balanced ${}_4\phi _3$’s.

#### Theorem 5.1

Let $$0<|q|<1$$, $$a,b,c,d,e,f,g,\frac{q^2abcd}{efg}\in {{\mathbb {C}}^*}$$, $$|efg|<|qbcd|$$. Then74

#### Proof

Start with (57), then notice that the ${}_8\!\Psi _8$ is invariant under the interchange $b\leftrightarrow c$. Then equating the right-hand sides under this replacement, making the replacements

$$ (a,b,c,d,e,f,g)\mapsto \left( \frac{aefg}{q^2c^2},\frac{fg}{qc},\frac{eg}{qc},\frac{ef}{qc},a,\frac{ab}{c},\frac{ad}{c}\right) , $$

and solving for the ${}_4\psi _4$ completes the proof. $\square$

One can obtain the following summation theorem by starting with Theorem 3.3, and setting $(d,f)=(1,a)$.

#### Corollary 5.2

Let $0<|q|<1$, $a,b,c,d,e\in {{\mathbb {C}}^*}$. Then one has the following summation theorem for a ${}_4\psi _4$ and two $_4\phi _3$’s namely75

#### Proof

Start with Theorem 3.3 and setting $d=1$ and $f=a$ then the ${}_8\!\Psi _8$ on the left-hand side becomes unity. Setting $g\mapsto d$ and simplifying completes the proof. $\square$

#### Remark 5.3

Note that there exists general transformations of a ${}_r\psi _r$ given in [5, Section 5.4] which are due to Slater [15]. For instance, taking [5, (5.4.5)], for $r=4$, this expansion is given by76such that $0<|q|<1$, $|z|<1$, $a,b,c,d,e,f,g,h\in {{\mathbb {C}}^*}$ with no vanishing denominator factors. However, these expansions do not seem useful for the purpose of obtaining summations or simplified transformations by taking limits. This is because the factors in Slater’s transformations involve modified theta functions that contain all parameters, such as the factor ${\vartheta }(\frac{abcdz}{qfgh};q)$ in (76), which makes it impossible to take confluent limits.

### Transformations for the special ${}_3\psi _3$ series

By starting with (62) and interchanging *d*, *f* and then *e*, *f* and then comparing the right-hand sides, we can obtain two transformations for a special ${}_3\psi _3$ series.

#### Theorem 5.4

Let $0<|q|<1$, $a,b,c,d,e,f\in {{\mathbb {C}}^*}$ such that $|def|<|qabc|$. Then7778

#### Proof

Notice that (62) is invariant under the interchange of the *b*, *c*, *d*, *e*, *f* variables. Equate the right-hand side of (62) with the same right-hand side with *d*, *f* interchanged. Making the variable replacements

79 $$\begin{aligned} (a,b,c,d,e,f)\mapsto \Biggl (\frac{def}{q^2c},\frac{ef}{qc},\frac{df}{qc},\frac{de}{qc},a,b\Biggl ) \end{aligned}$$

produces (77). Equating the right-hand side of (62) with *d*, *f* interchanged with the same right-hand side with *e*, *f* interchanged and making the identical variable replacements (79) produces (78), which completes the proof. $\square$

#### Remark 5.5

If one sets $d=q$ in (77), (78), or $e=q$ in (77), then the second terms on the right-hand sides vanish. This then produces the two-term nonterminating transformations for a special ${}_3\phi _1$ [5, (III.9), (III.10)]. On the other hand, if one sets $e=q$ in (78) or $f=q$ in (77), (78), then the special ${}_3\psi _3$ on the left-hand side becomes a special ${}_3\phi _2$ and the ${}_3\psi _3$ on the right-hand sides become a specialized ${}_3\psi _3$. For instance, for the $e=q$ in (78) case, solving for this specialized ${}_3\psi _3$ replacing variables produces the following transformation80There will be other specialized transformations of a ${}_3\psi _3$ if one sets $f=q$ in (77), (78). We leave these transformations to the reader.

#### Corollary 5.6

Let $0<|q|<1$, $a,b,c,d,e,f\in {{\mathbb {C}}^*}$ such that $|def|<|qabc|$. Then81

#### Proof

Start with Theorem 5.1 and take the limit as $d\rightarrow \infty$. The limit of the last term on the right-hand side of (74) vanishes. The limit of the third to last term on the right-hand side of (74) becomes the last term on the right-hand side of (81). The limits of the two ${}_8\!W_7$’s become ${}_7\!W_6^1$’s which are evaluated as special ${}_3\phi _2$’s using (24). Using the theta function addition formula (6) completes the proof. $\square$

#### Remark 5.7

One of the referees inquired whether Corollary 5.6 might be connected with [5, (5.4.4)] as a ${}_3\psi _3$ with the required argument. Performing the necessary substitutions leads to the following symmetric sum of three terms, namely:82Corollary 5.6 and (82) might very-well be related and there is a lot of freedom to work here because the nonterminating ${}_3\phi _2$’s with non-*q* argument satisfy the transformations given by [5, (III.9), (III.10), (III.34)]. However, we were unable to find a direct route which relates both of these transformation formulas.

#### Remark 5.8

If one takes the limit as either $d\rightarrow q$ or $e\rightarrow q$ in Corollary 5.6, then two of the terms on the right-hand side vanish leading to trivial identities. Alternatively, if one takes the limit as $f\rightarrow q$ in Corollary 5.6, then one of the terms on the right-hand side vanishes leading to the following transformation formula for a special nonterminating ${}_3\phi _2$, namely83The above transformation formula can be verified by starting with the three-term transformation formula [5, (III.33)] which is invariant under the replacement of the variables *d*, *e*. Performing this variable interchange and solving for the second term on the right-hand side obtains two identities which can then be multiplied by the corresponding infinite *q*-shifted factorials in (83) and then subtracted. The resulting expression then simplifies to the correct result.

### Transformations for a ${}_2\psi _3$ series

By starting with (69) and taking advantage of the symmetry in the parameters *b*, *c*, *d*, *e*, one can obtain a transformation of a special ${}_2\psi _3$.

#### Theorem 5.9

Let $$0<|q|<1$$, $$a,b,c,d,e\in {{\mathbb {C}}^*}$$. Then8485

#### Proof

Start with (69) and compare the two invariant terms with *d* and *e* replaced which are equal. Cancel like terms, and solving for the first ${}_2\psi _3$ produces (84). To obtain (85), start with with (77) and take the limit as $b\rightarrow \infty$. In both cases, the second term on the right-hand side vanishes in this limit and the ${}_3\psi _3$ on the right-hand side becomes a ${}_2\psi _2$. This completes the proof. $\square$

### Transformations for the ${}_2\psi _2$ series of arbitrary argument

#### Corollary 5.10

Let $0<|q|<1$, $a,b,c,d,z\in {{\mathbb {C}}^*}$, such that $|cd/(abz)|<1$, $|z|<1$. Then one has the following transformation of a ${}_2\psi _2$ in terms of a sum of a ${}_3\psi _3$ and a ${}_3\phi _2$ with arguments *q* and with one vanishing denominator element, and a very-well-poised ${}_6\Psi _6^2$, namely8687

#### Proof

Starting with (66) (which is (87)) and equating it with (65) while replacing $\{a,b,c,d,e\}\mapsto \left\{ {abz}/{q},a,b,{qa}/{c},{qa}/{d}\right\}$ completes the proof. $\square$

#### Remark 5.11

Note that setting $d=q$ in (86) converts it to (21).

#### Remark 5.12

The transformation which describes the parameter interchange symmetry in the above ${}_2\psi _2$ is (35).

### Transformation for a ${}_1\psi _3$ series

We can obtain a transformation for a ${}_1\psi _3$ by starting with transformations of a ${}_2\psi _3$ and taking the limit as one of the numerator parameters goes to infinity.

#### Theorem 5.13

Let $$0<|q|<1$$, $$a,b,c,d\in {{\mathbb {C}}^*}$$. Then8889

#### Proof

Start with (84) or (78) and let $a\rightarrow \infty$. In both cases the limit leads to the same transformation, which completes the proof. $\square$

### Summation for a ${}_0\psi _1$ series

Taking the limit $b\rightarrow 0$ in Corollary 4.6 converts the ${}_2\psi _2$ to a ${}_1\psi _1$ which is summable using Ramanujan’s ${}_1\psi _1$ summation which produces the following summation result. In fact, the following result is just a confluent limit of Ramanujan’s ${}_1\psi _1$ summation (33), namely replacing *z* by *z*/*a*, taking $a\rightarrow \infty$ and replacing *b* by *a*.

#### Corollary 5.14

Let $$0<|q|<1$$, $$a,b,c,z\in {{\mathbb {C}}^*}$$. Then90

#### Proof

Start with Corollary 4.6 and take the limit $b\rightarrow 0$ which converts the ${}_2\psi _1^1$ to a ${}_1\psi _1$, then replace $c\mapsto b$. The ${}_1\psi _1$ can be summed using Ramanujan’s ${}_1\psi _1$ summation (33). Solving for the ${}_0\psi _1$ replacing $\{az,b\}\mapsto \{z,a\}$ and canceling common factors completes the proof. $\square$

### Summation for the ${}_0\psi _0^2$ series

#### Corollary 5.15

Let $$0<|q|<1$$, $$z\in {{\mathbb {C}}^*}$$. Then91

#### Proof

Start with Corollary 4.8 and take the limit as $b\rightarrow \infty$. The limit of the ${}_1\psi _0^2$ is the ${}_0\psi _0^2$. Note that the triple-product identity (5), given by92If one replace $q\mapsto q^2$, then we obtain93This completes the proof. $\square$

## Data Availability

The authors confirm that all the data supporting the findings of this study are either referred to in the list of references or are available within the article.

## Footnotes

1 While it is a routine matter to *formally* take the limit $q\rightarrow 1^{-}$ (and is useful to *find* the desired result), a rigorous justification requires an explanation why taking the term-wise limit in the summands of the series is allowed. This typically requires that the series is majorized by a convergent series not depending on *q* and follows the arguments provided by Koornwinder in [10, Appendix A].
